# Characterization of Densified Pine Wood and a Zero-Thickness Bio-Based Adhesive for Eco-Friendly Structural Applications

**DOI:** 10.3390/ma16227147

**Published:** 2023-11-13

**Authors:** Shahin Jalali, Catarina da Silva Pereira Borges, Ricardo João Camilo Carbas, Eduardo André de Sousa Marques, João Carlos Moura Bordado, Lucas Filipe Martins da Silva

**Affiliations:** 1 Institute of Science and Innovation in Mechanical and Industrial Engineering (INEGI), Rua Dr. Roberto Frias, 4200-465 Porto, Portugal; 2 Departamento de Engenharia Mecânica, Faculdade de Engenharia, Universidade do Porto, Rua Dr. Roberto Frias, 4200-465 Porto, Portugallucas@fe.up.pt (L.F.M.d.S.); 3 Centro de Recursos Naturais E Ambiennte (CERENA), Instituto Superior Técnico, University of Lisbon, 1049-001 Lisbon, Portugal; jcbordado@tecnico.ulisboa.pt

**Keywords:** bio-based adhesive, densified wood, pine wood, sustainable adhesive joints, zero-thickness adhesive

## Abstract

This study investigates a sustainable alternative for composites and adhesives in high-performance industries like civil and automotive. This study pioneers the development and application of a new methodology to characterize a bio-based, zero-thickness adhesive. This method facilitates precise measurements of the adhesive’s strength and fracture properties under zero-thickness conditions. The research also encompasses the characterization of densified pine wood, an innovative wood product distinguished by enhanced mechanical properties, which is subsequently compared to natural pine wood. We conducted a comprehensive characterization of wood’s strength properties, utilizing dogbone-shaped samples in the fiber direction, and block specimens in the transverse direction. Butt joints were employed for adhesive testing. Mode I fracture properties were determined via compact tension (CT) and double cantilever beam (DCB) tests for wood and adhesive, respectively, while mode II response was assessed through end-loaded split (ELS) tests. The densification procedure, encompassing chemical and mechanical processes, was a focal point of the study. Initially, wood was subjected to acid boiling to remove the wood matrix, followed by the application of pressure to enhance density. As a result, wood density increased by approximately 100 percent, accompanied by substantial improvements in strength and fracture energy along the fiber direction by about 120 percent. However, it is worth noting that due to the delignification nature of the densification method, properties in the transverse direction, mainly reliant on the lignin matrix, exhibited compromises. Also introduced was an innovative technique to evaluate the bio-based adhesive, applied as a zero-thickness layer. The results from this method reveal promising mechanical properties, highlighting the bio-based adhesive’s potential as an eco-friendly substitute for synthetic adhesives in the wood industry.

## 1. Introduction

In recent years, there has been a growing interest in the development of sustainable alternatives to fossil-fuel-based products. As part of these endeavors, bio-based materials have emerged as a promising solution to mitigate the environmental impact across various industries. Among these applications, the production of load-bearing structures stands out, wherein natural fibers from plants, such as flax, jute, and palm trees, can be integrated into composite materials to create eco-friendlier alternatives. Another approach involves the utilization of natural composite materials directly. Wood, in particular, has been used for various purposes throughout history due to it being a natural and renewable resource. Its durability, environmental resistance, mechanical strength, low weight, flexibility in shaping, abundance in many geographic regions, and reasonable cost make it a desirable material [1,2,3]. 

Venkatesan et al. [4] prepared biodegradable composites from poly(butylene adipate-co-terephthalate) (PBAT) and carbon nanoparticles. They characterized these composites in terms of morphology, thermal stability, mechanical properties, and biodegradability. They found that the composites showed improved thermal and mechanical performance compared to pure PBAT, and also exhibited good biodegradability under composting conditions. In another study [5], the authors prepared nanocomposite films from PBAT and zinc oxide nanoparticles, determining the mechanical, thermal, and biological properties of the films. They found that the films had good antimicrobial activity against *E. coli* and *S. aureus*.

Wood is a natural composite material that is susceptible to stress concentrations and notches, making traditional joining methods, such as riveting and bolting, unsuitable. Polymers from liquefied biomass were synthesized and used as wood adhesives. The bio-based polymers exhibited comparable or superior performance to petroleum-based adhesives in terms of bond strength, thermal stability, and water resistance [6].

Adhesive bonding is often seen as a better option as it offers a larger and more uniform bonded area without introducing stress concentrations [7,8]. However, the joint’s failure load and mode depend on the combined properties of the adhesive and substrates [9]. When bonding wood substrates, the peel loading can cause delamination between different grain plies, resulting in complete joint failure. To prevent this failure mode, recent techniques, such as densification, substrate toughening, and physical modifications of the adhesive have been proposed [10,11]. Additionally, working with wood substrates can present challenges in terms of mechanical characterization and consistency [12,13], due to wood being a complex and heterogeneous material, with properties that can vary depending on factors such as species, growth conditions, grain slope and size, defects, knots, shakes (cracks and notches of the wood), and age. This can make it difficult to develop standardized testing protocols to ensure a consistent performance in different applications [14,15]. To overcome these problems, wood densification processes can be used to alter the failure mode of this material, improving result consistency. Other methods, like using wood particles or laminates, and wooden composites also can help reduce these design challenges since the material properties can be made to be more uniform. This is particularly important to achieve a predictable behavior in structural applications [16,17,18].

Additionally, the mechanical properties of hemicellulose and lignin, which are key components of wood, change significantly with service temperature, depending on whether they are above or below their glass transition temperature (*T_g_*). When dry, the *T_g_* of lignin ranges from 134–235 °C, while the *T_g_* of hemicellulose ranges from 167–217 °C. Since the service temperature is mostly below the *T_g_*, these values are generally not a design concern. However, *T_g_* has been observed to significantly decrease as moisture content increases. A decrease in *T_g_* can be problematic as it can lead to reduced mechanical strength, dimensional instability, and increased susceptibility to deformation or failure. It can also affect thermal stability, promoting creep and relaxation at elevated temperatures. Additionally, a lower *T_g_* can accelerate chemical reactions, causing degradation and reducing the material’s long-term durability. Moisture acts as a plasticizer that weakens the secondary bond between polymer chains, leading to increased flexibility of the molecules. Proper moisture management is crucial for successful wood densification and preserving structural integrity [19,20,21]. The sensitivity of wood to moisture leads to a phenomenon called set-recovery, which has implications for both the absorbed energy in calluses and the covalent bonds between polymeric chains.

Set-recovery in wood refers to its ability to partially regain its original shape and dimensions after undergoing deformation or stress. This behavior has an impact on the energy-absorption capacity of calluses (scar tissue) formed within the wood and the strength of the covalent bonds holding the polymer chains together. Sadat Nezhad et al. [22] employed thermo-hydro-mechanical (THM) methods to increase wood density and observed a set-recovery of approximately 44% after three wet–dry cycles. Additionally, Laine et al. [20] reported a set-recovery of around 60% after saturating densified wood samples with water, which was later reduced to nearly zero through the application of thermal modification post-compression [23].

To address the problem of set-recovery while achieving greater levels of densification, chemical pre-treatments have been utilized [18]. These treatments are based on the delignification process used in the paper industry, which can be adapted to modify wood properties. By removing lignin and hemicellulose, the resulting densified wood exhibits changes in mechanical properties. While the passage does not provide specific details, it suggests that the removal of lignin decreases the stiffness of the wood in the transverse direction. However, the overall effect on stiffness and strength depends on factors such as wood species, processing conditions, and the degree of densification. Additionally, the physical interlocking of cellulose fibers within the densified wood structure enhances its mechanical strength by providing structural support and resistance to deformation. The specific mechanical properties achieved through this treatment process will vary depending on the intended application and desired characteristics [22,23,24].

Recently, the drive for more sustainable bonded structures has led to the development of various structural and non-structural bio-based adhesives and the adaptation and characterization of many bio-based polymers, which are natural, renewable, and non-petroleum-based, for use in diverse applications. However, the number of materials that can fulfil this role is limited. Tannin, lignin, carbohydrates, unsaturated oils, proteins, and protein hydrolysates are some of the natural materials that have been used as adhesives with good results. In addition, dissolved wood and wood welding with self-adhesion have also been presented as potential alternatives to bonding [25,26,27,28].

The current study focuses specifically on natural-oil-based polyurethane adhesive, a bio-based adhesive known for its advantageous properties. Oil-based polyurethane adhesives have shown great potential in providing strong and durable bonds, while also possessing eco-friendly characteristics.

Depending on the polar urethane group employed, a wide range of adhesive behaviors, ranging from rubber-like elasticity to brittle–hard characteristics, can be achieved [29]. These adhesives can be categorized as one-component or two-component systems. Two-component polyurethane adhesives consist of separate isocyanate and polyol components that are mixed prior to application, offering faster curing rates and unlimited depth of cure. In contrast, one-component polyurethane adhesives are prepolymers containing isocyanate groups that react with moisture in the air or on the substrate to cure, eliminating the need for mixing equipment but having limitations in depth of cure [27]. Several recent studies have examined the influence of various factors on the properties of moisture-cured polyurethane (PU) adhesives, shedding light on their potential applications.

This study develops and applies a new methodology to characterize a bio-based zero-thickness polyurethane adhesive that uses 70% natural resources as raw materials. This method allows for the accurate measurement of the strength and fracture properties of the adhesive under zero-thickness conditions. The study also characterizes the densified pine wood, a novel wood product with enhanced mechanical properties, and compares it with natural pine wood. The main contributions of this work are the advancement of the knowledge on bio-based adhesives and densified wood products, and the demonstration of their superior performance over conventional materials. To understand the wood’s mechanical properties, dogbone-shaped samples were used for the fiber direction, while block specimens were employed for the transverse direction. Fracture properties were determined through testing compact tension (CT) and end-loaded split (ELS) specimens. For the bio-based adhesive, tensile properties were obtained using butt joints with a wooden substrate, and fracture properties were measured using double cantilever beam (DCB) and ELS joints. This study aims to assess the potential of densified pine wood and the zero-thickness bio-based adhesive as sustainable alternatives by comprehensively characterizing their mechanical properties. Given the focus on structural applications, established processes and testing procedures commonly associated with structural adhesives were employed. These techniques transcend those typically used for low-strength or wood adhesives. In this context, the tests yield precise material properties, underpinned by rigorous finite element analysis. This analytical approach enables accurate modeling of the tests, ensuring that the obtained properties align with the loads acting on the adhesive layer.

## 2. Materials

### 2.1. Wood

#### 2.1.1. Natural Pine Wood

In this study, Pinus pinaster wood (pine wood) sourced from the Alentejo region in South Portugal was used as the main material. Pine wood was chosen as the main material for various reasons, which include its wide availability, low cost, and good mechanical qualities, such as durability, stiffness, and strength. The wood samples were extracted from trees that were 15 years old and located in the coastal area of the region. The age and location of the wood samples are important factors that affect the quality and characteristics of the wood, as they influence its density, moisture content, mechanical properties, and durability. The precise origin of the wood samples was considered in this study, as it can have a significant impact on the performance of the wood-based products. The wood samples were selected based on the criteria proposed by Moura et al. [15], who studied the properties of Pinus pinaster wood from the same regions of Portugal.

**Figure 1:**
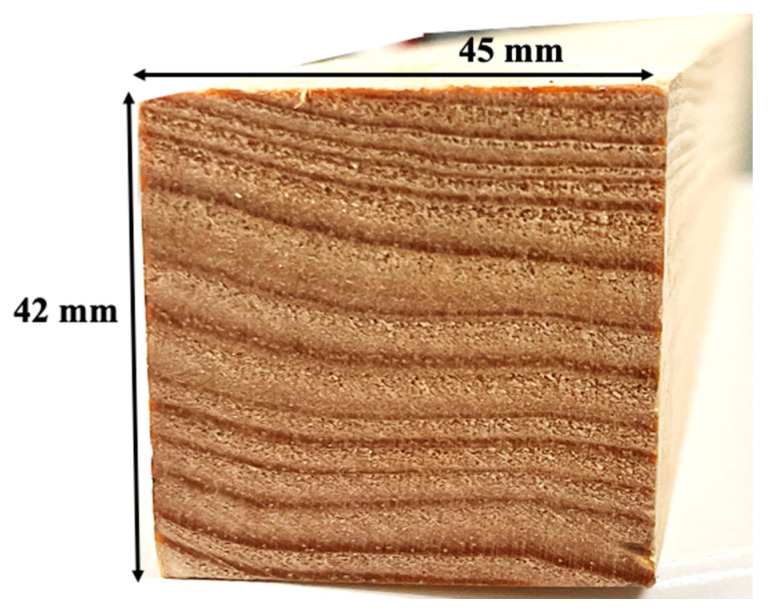
Wood timber dimensions.

The geometries and dimensions of the pine wood blocks, used for both pine wood characterization and the densification process, are shown in Figure 1. As represented, the wood had the rings as parallel as possible to one of the sides of the timber. The initial length of the timber was 1 m, and smaller pieces were cut to the required dimensions for the tests. Pine wood was chosen for its availability, affordability, and favorable mechanical properties, including strength, stiffness, and durability. The elastic constants and strength properties have to be determined in the longitudinal (L), radial (R), and tangential (T) directions. Previous work by Oliviera et al. [30] fully mechanically characterized this type of natural pine wood, and the summarized results are presented in Table 1.

#### 2.1.2. Densified Pine Wood

The blocks used for wood densification were cut from natural pine wood into pieces measuring 45 × 40 mm, with an average length of 240 mm.

**Figure 2:**
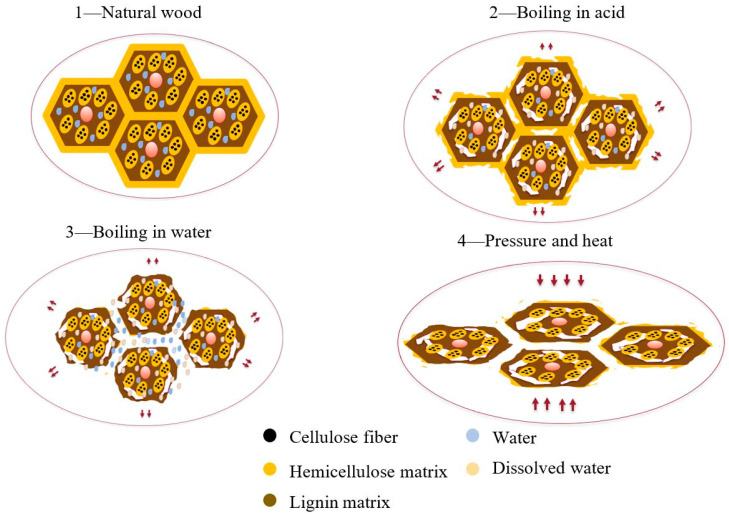
Simplified scheme illustrating the modification of wood’s cellular structure through a delignification process involving a chemical reaction, followed by a compression stage.

The densification process was based on the method developed by Song et al. [31] and involved two main steps, as in Figure 2. During the first step, wood blocks were boiled in a chemical bath containing a solution of 2.5 M NaOH and 0.4 M Na_2_SO_3_ for seven hours, allowing the chemical catalyst to penetrate the cell walls and increase cell volume. This resulted in the destruction of hemicellulose and lignin matrices. Subsequently, the blocks were boiled in deionized water for an hour to remove the catalyst, with the reaction continuing until complete elimination. To ensure thorough chemical removal, the deionized water was changed every 30 min. Water absorption during this step further increased cell volume, creating empty spaces between the cells filled with water. The second step of the densification process involved a thermo-mechanical procedure. The wood blocks were placed in a hot-press for 24 h under a pressure of 3 MPa and 100 °C using a steel mold developed in a previous study [32], to compress and deform the cell walls. This caused the collapse of the cell walls without damaging the fibers, resulting in increased density and strength. Maintaining precise humidity levels was paramount throughout the production of both pine wood and densified pine wood. Typically, wood undergoes conditioning to achieve a moisture content ranging between 12% and 20%. This is done by exposing the wood to controlled humidity and temperature conditions for a certain period of time. For densified pine wood, maintaining moisture content within this specified range was of utmost importance. This ensures that the material preserves its intended strength, flexibility, and dimensional stability. Deviations from these critical moisture levels could cause issues such as warping, cracking, or a decline in mechanical properties, all of which could significantly affect the quality and performance of the final product. The moisture content range of 12% to 20% was chosen based on the expected service conditions of the densified pine wood products, as well as the recommendations from previous studies on wood densification. To further curtail moisture content of densified wood and align it with that of pine wood, a meticulous process was employed. Therefore, densified wood blocks were kept in silica gel (with a diameter of 2–5 mm with moisture indicator changing the color), maintained at a temperature of 70 °C for a duration of 48 h. This method harmonized the moisture content, enhancing both durability and resistance to chemical reactions.

### 2.2. Bio-Based Adhesive

A polyurethane bio-based adhesive, derived from 70% of renewable biomass sources, such as vegetable oils according to the ASTM D6866 standard, was characterized. This adhesive was designed for strong adhesion to wood which is a prototype product, developed by the team of Professor João Bordado at Instituto Superior Técnico. It is not yet commercially available, but it shows potential as a sustainable alternative to synthetic adhesives. It is produced in an irreversible reaction, without humidity, in a reactor under a nitrogen atmosphere, and heating is done with a thermal oil coil. It uses an aliphatic isocyanate as a basis, which contains 70% plant matter, which are more easily biodegradable. Manufacturing the bio-adhesive is estimated to consume 15 to 20% less energy than those derived from petroleum. The bio-adhesive contains pentamethylene diisocyanate and polyisocyanate, which react with the hydroxyl (OH) groups in the wood substrate. This reaction creates strong bonds, and, therefore, increasing the humidity of the substrates could speed up the curing process. To ensure uniform curing of the bio-adhesive in the joints, it was important to keep consistent moisture levels across all samples. The synergistic interplay between the bio-adhesive and the wood’s OH groups, along with the influence of humidity, brings about multiple benefits. Not only does it enhance mechanical interlocking, bolstering the physical and mechanical bonds within the joints, but it also facilitates superior chemical bonding. High-strength oak wood was used as the substrate for reliable testing. Curing bulk samples was challenging due to the zero-thickness bond requirement. Also, the absence of adhesive thickness was confirmed through the bonding of substrates directly to each other by applying pressure without using any spacer, thereby ensuring a ‘zero-thickness’ condition. The adhesive undergoes an initial curing phase at 100 °C for 8 h, followed by a recommended 48 h curing period at room temperature, as suggested by the developer. This approach ensures that the curing conditions are in line with the recommendations of the data sheet.

## 3. Experimental Details

The mechanical tests described in this section were conducted under quasi-static conditions, using a uniaxial universal testing machine (Instron 3367, united states of America based) with a load cell capacity of 30 kN and a displacement rate of 1 mm/min. For each condition, at least three specimens were tested, and also the dimensions of all tested specimens were controlled using a caliper with an accuracy of 0.1 mm.

### 3.1. Characterization of the Natural Pine Wood and Densified Pine Wood

#### 3.1.1. Density Measurement

To evaluate the effect of the wood densification procedure on the actual densification of wood, the volume of each wood block when natural and densified was measured, as well as its mass. The dimensions were measured using a caliper with an uncertainty of 0.05 mm and the mass using a digital scale with an uncertainty of 0.01 g. The density was then determined by calculating the quotient between the mass and the volume of the block.

#### 3.1.2. Strength Tests

**Bulk tensile test**

**Figure 3:**
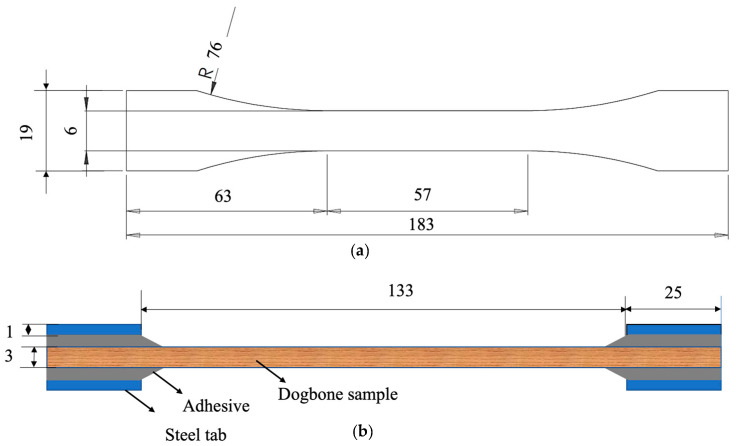
Geometry of wooden dogbone specimen (**a**), and attachment of tabs for testing (**b**). (Dimensions in mm).

To assess the strength along the fiber direction, dogbone-shaped samples were manufactured for both natural pine wood and densified wood, as shown in Figure 3a. Reduced scale specimens were used due to the geometric restrictions of the densified wood block. These specimens were validated against standard specimens of pine wood, comparing with the standard specimen results of Moura et al. [15]. To prevent sample failure at the grips, 1 mm thick steel tabs were bonded to both ends of the samples. Adhesive fillets were also applied to ensure a more uniform stress transfer to the gage length of the specimens; as depicted in Figure 3b, five specimens were tested.

**Block specimen test**

**Figure 4:**
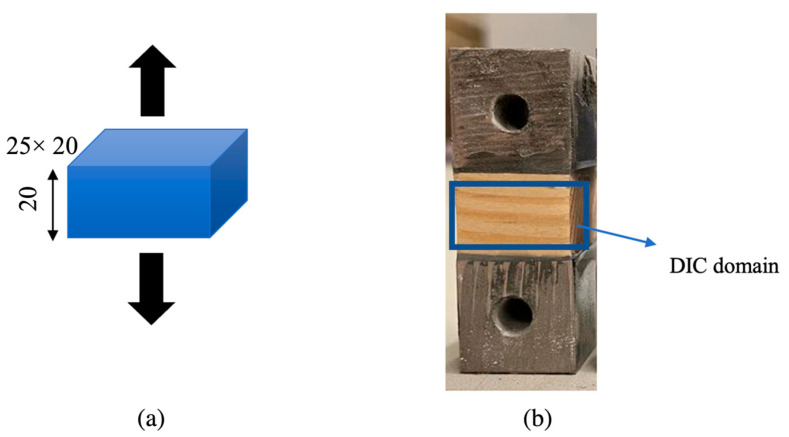
Block specimen geometry and dimensions (**a**), and digital image correlation (DIC) domain (**b**). (Dimensions in mm).

To determine the strength of the wood in the direction parallel to the fibers, blocks of wood and densified wood were cut to dimensions of 20 × 25 × 20 mm. In order to mount the wood in the testing machine, steel blocks (shown in Figure 4) were bonded to the wood using Araldite AV138, an epoxy adhesive. The adhesive was cured for 24 h at room temperature. Once cured, the adhesive fillets were carefully cleaned using sandpaper to ensure accurate measurements. To minimize any potential influence of the adhesive and steel specimens on the measurements, the strain field of all samples was obtained using digital image correlation (DIC); four specimens were tested. This helped in accurately assessing the properties of the wood without interference from the bonding materials.

#### 3.1.3. Fracture Tests

**Compact tension test (CT)**

**Figure 5:**
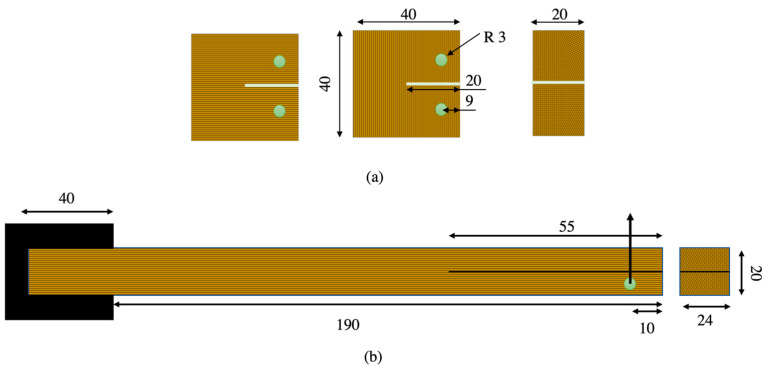
Representation of the test specimens used to conduct compact tension (CT) tests (**a**), and end-loaded split (ELS) tests (**b**). (Dimensions in mm).

CT specimens were manufactured to determine the mode I fracture energy of wood. The CT test is commonly used to determine the fracture toughness of brittle materials such as wood. These CT specimens were designed with a centrally located crack in both the fiber and transverse directions, which was loaded in tension to create a pure mode I loading. The dimensions of the CT specimens are presented in Figure 5a. Six specimens were tested to ensure the repeatability of the results.

**End-loaded split test (ELS)**

ELS specimens were produced to determine the mode II fracture energy of the wood. Similar to the CT specimens, the ELS specimens were designed with a centrally located crack, which was loaded in a transverse direction to achieve a pure mode II condition. Due to the geometrical constraints of the densified wood block, the length of ELS specimens in the fiber direction was limited to 230 mm. Three specimens were tested to ensure the repeatability of the results. The dimensions of the ELS specimens are shown in Figure 5b. Mode II fracture energy is the energy required to propagate a crack perpendicular to the direction of the applied load. To calculate the fracture energy from the load displacement curves, the compliance-based beam method (CBBM) [33] was chosen as the preferred data-reduction approach. CBBM [33] was used to determine the fracture energy without the need for measuring crack propagation during testing. Additionally, CBBM takes into account the fracture process zone (FPZ) formed ahead of the crack tip allowing for the calculation of a corrected or equivalent crack length (*a_eq_*). In this study, load-displacement data obtained from the universal tensile test machine were used to compute the fracture energy using CBBM.

### 3.2. Characterization of Bio-Based Adhesive

This study focused on characterizing a prototype adhesive that relies on the moisture in wood substrates for curing, necessitating a zero-thickness bond. To ensure that failure occurred only in the adhesive and not the wood, stronger oak wood was used. Nonetheless, surface preparation was critical, involving polishing with 400-grade sandpaper for a smooth, uniform surface. Compressed air was used to remove dust particles that could hinder effective bonding, and acetone was applied for thorough cleaning, removing any contaminants.

#### 3.2.1. Strength Tests

**Butt-joint test**

**Figure 6:**
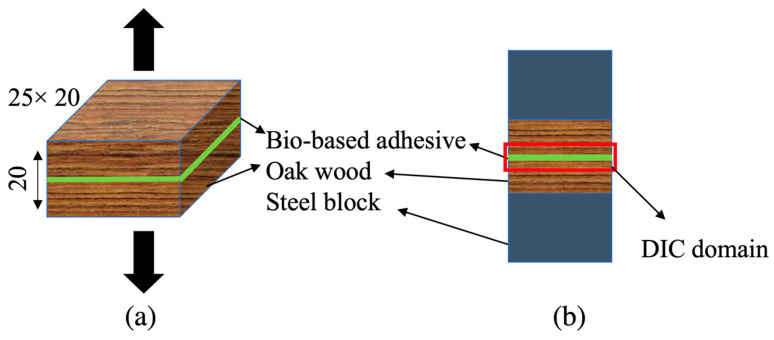
Butt-joint geometry and dimensions (**a**); considered digital image correlation (DIC) domain (**b**). (Dimensions in mm).

To measure the strength properties of the bio-based adhesive, wood butt joints were used. To prepare these joints, oak wood with an area of 20 × 25 mm was cut with the thickness of 10 mm (Figure 6a). After the surface preparation described above, the bio-based adhesive was applied on the surfaces of both the substrates. The substrates were then bonded to each other carefully, to avoid any misalignments, and pressure was applied to ensure even contact between the wood substrates, using a clamp. The adhesive was cured and steel blocks to allow for testing were bonded as described for the wood block specimens.

DIC was used to measure strain near the bondline (Figure 6), through a speckle pattern introduced to the bonded area. This approach eliminated additional elongation caused by the wood, resulting in a precise determination of the bio-based-adhesive strain.

**Thick adherend shear test (TAST)**

**Figure 7:**
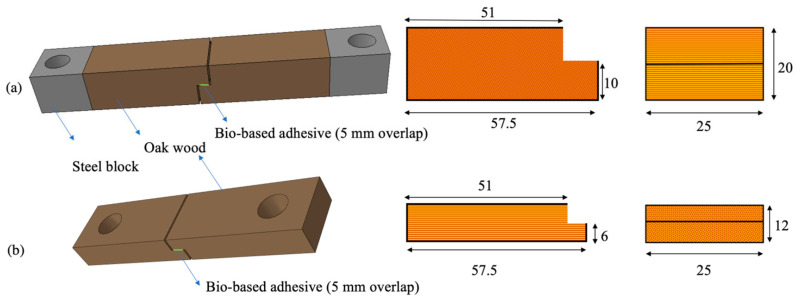
Thick adherend shear test (TAST) specimen geometries with the wood loaded in the transverse (**a**) and fiber direction (**b**). (Dimensions in mm).

The TAST was used to assess the shear properties of the adhesive. The testing setup consisted of two different joint configurations, designed to explore variations in substrate geometry and thickness. The two joints are shown in Figure 7a,b. The first joint, shown in Figure 7a, featured a thicker substrate loaded perpendicular to the grain direction, while the second joint, shown in Figure 7b, employed a slightly thinner substrate, loaded in the grain direction.

#### 3.2.2. Fracture Tests

**Double cantilever beam test (DCB)**

**Figure 8:**
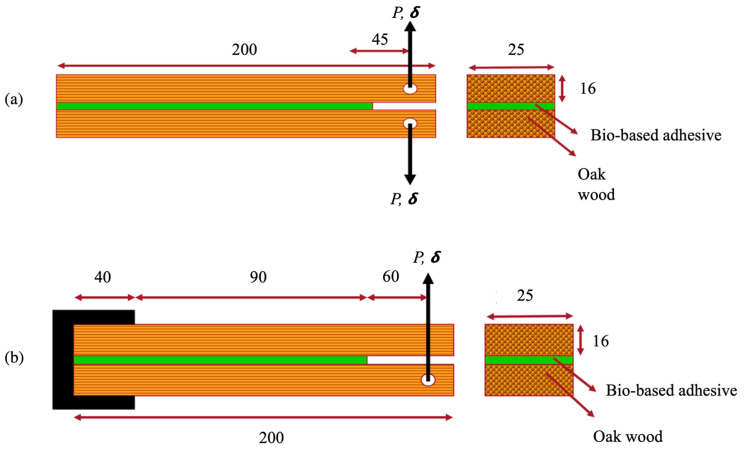
Representation of the test specimens used to conduct double cantilever beam test (DCB) tests (**a**) and end-loaded split (ELS) tests (**b**). (Dimension in mm).

To determine the mode I fracture energy of the bio-based adhesive, DCB specimens were used. Following the surface-preparation procedures, the bio-based adhesive was applied to both sides of the joint. In order to introduce a pre-crack of 45 mm, a 0.1 mm thick Teflon film was placed between the two substrates. To ensure uniformity in the joint-preparation process, a total of four clamps were employed, applying controlled pressure over the entire bondline. The testing process incorporated specimens with specific dimensions and geometries, as depicted in Figure 8a. To calculate the fracture energy of the bio-based adhesive under quasi-static conditions, CBBM [33] was utilized.

**End-loaded split test (ELS)**

To investigate the mode II fracture energy of the bio-based adhesive, ELS specimens were employed in the experimental procedure. The manufacturing process followed the same set of procedures used for the DCB, since it has the same joint geometry. For the ELS joints, a pre-determined pre-crack length of 60 mm was established. The specimen geometry and the testing procedure are shown in Figure 8b.

## 4. Results and Discussion

### 4.1. Characterization of the Wood and Densified Wood

#### 4.1.1. Density Measurement

**Figure 9:**
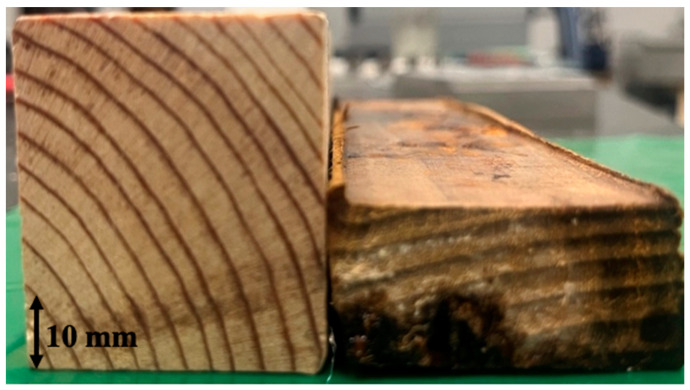
Wood beam before (**left**) and after (**right**) densification.

The pine wood displayed a substantial increase in its average density, changing from 0.56 ± 0.03 g/cm^3^ to 1.23 ± 0.12 g/cm^3^ (Figure 9). These findings demonstrate a successful enhancement of the wood’s density through the densification process.

#### 4.1.2. Strength Tests

**Figure 10:**
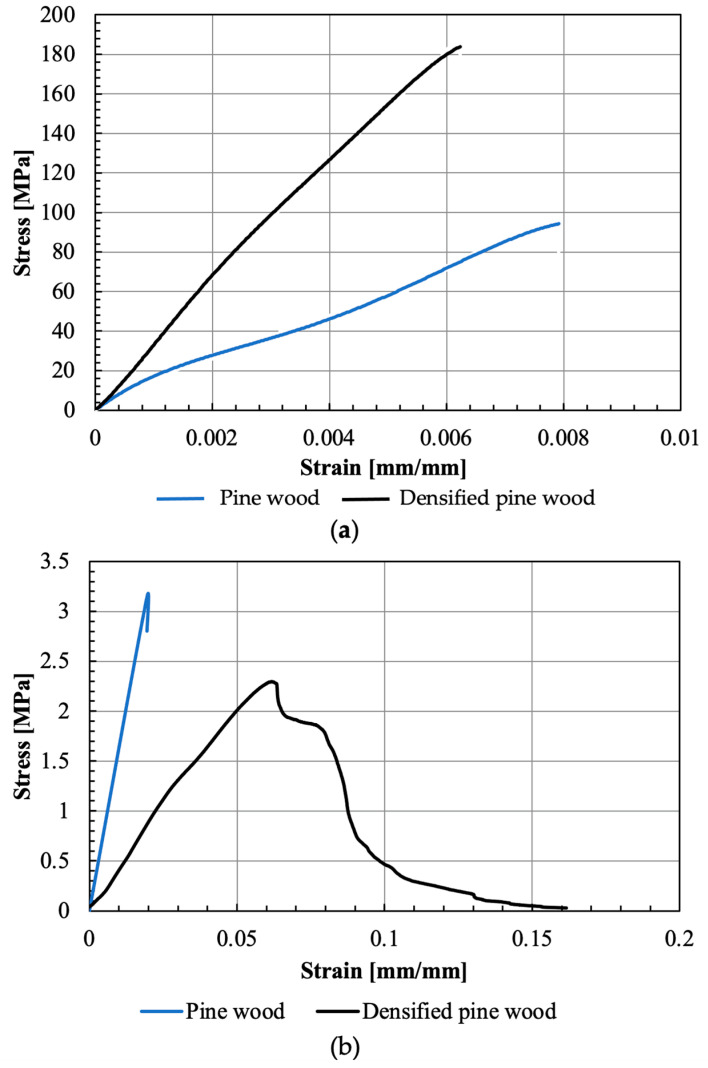
Representative tensile stress–strain curve of wood and densified wood in the tensile (**a**) and transverse (**b**) directions.

Figure 10 illustrates a representative stress–strain curve obtained from the manufactured samples of both pine wood and densified pine wood for strength properties. The determined values of Young’s modulus and strength in the fiber and transverse direction can be seen in Table 2.

In the case of pine wood, the results for natural pine wood were in line with the study by Moura et al. [15], that reported a Young’s modulus and strength of 12 GPa and 97.5 MPa, respectively. This validates the reduced-scale dogbone specimens used. Regarding transverse properties, Moura et al. [15] registered a strength of 4.2 MPa, slightly higher than the values obtained with the block specimens used in this study.

Densified pine wood displayed a significant increase in tensile strength and stiffness, due to the significant increase in the volume fraction of the fibers in the wood. However, unlike what was found for the properties in the fiber direction, the densification process resulted in a decrease in the transverse properties of pine wood, as the wood matrix (responsible for the transverse strength) was degraded during this process.

#### 4.1.3. Fracture Tests

**Figure 11:**
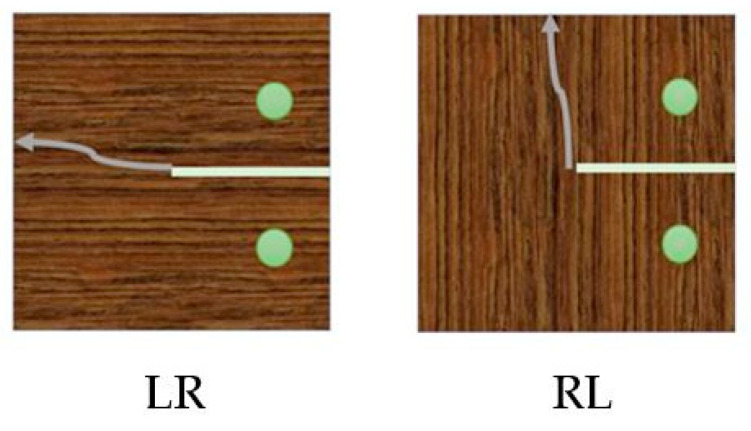
Crack propagating along the grains perpendicular (LR) and parallel to the loading direction (RL).

The fracture toughness for natural pine wood and densified pine wood obtained are presented in Table 3. For the CT tests, it was seen that, both in the fiber direction and the transverse direction, the crack propagated between the grains of wood, as shown in Figure 11. This demonstrates that the densification process significantly enhances the fracture properties of wood.

**Figure 12:**
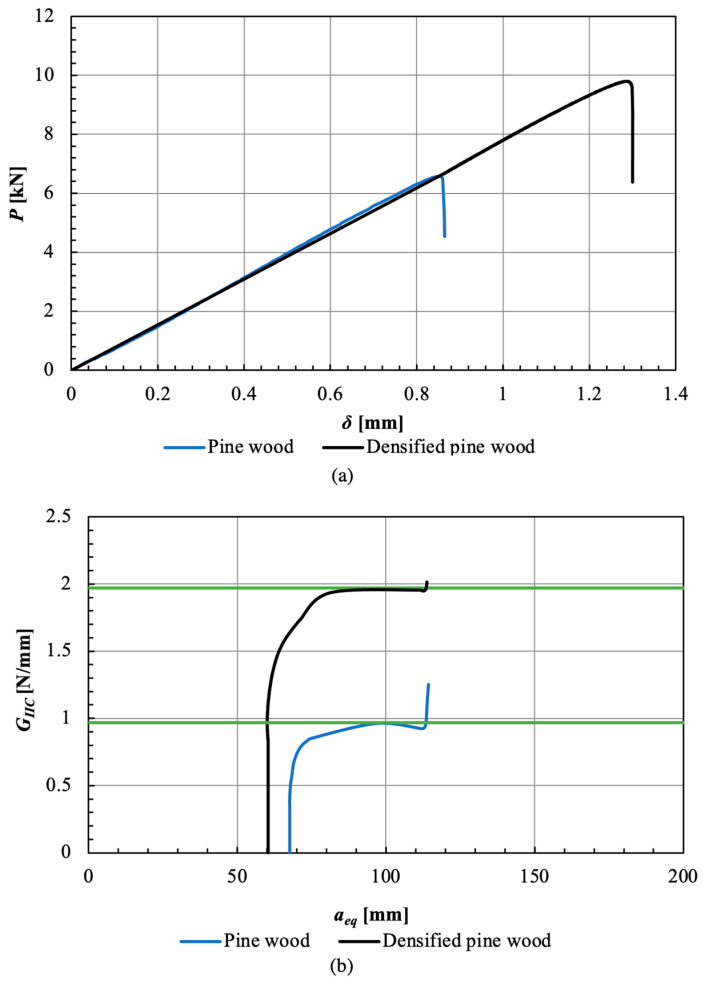
ELS test results for the woods: P-δ curve (**a**) and R-curve (**b**). Green lines presented the obtained fracture energy.

Mode II fracture energy of densified wood was measured by testing ELS specimens, with CBBM being used to generate an R-curve from a *P-δ* curve. The average mode II fracture energy of pine wood and densified pine wood was about 0.9 ± 0.1 N/mm, and 1.7 ± 0.2 N/mm, respectively. Typical behavior of ELS specimens is shown in the *P-δ* curve ELS in Figure 12a and the obtained R-curve is presented in Figure 12b.

The densification process increases wood density, leading to improved stiffness, strength, and dimensional stability. The alignment of fibers during densification further enhances mechanical properties, making it suitable for structural applications. In the fiber direction, densified wood showed a 90% increase in Young’s modulus and an 85% increase in strength, while transverse properties decreased due to lignin- and wood-matrix destruction. However, overall, densified wood remains still appear to be a valid option for structural applications and a sustainable alternative to traditional materials. Finally, the fracture properties of densified wood exhibited higher resistance to failure, with significant improvements in fracture toughness and energy.

### 4.2. Characterization of Bio-Based Adhesive

#### 4.2.1. Strength Tests

**Figure 13:**
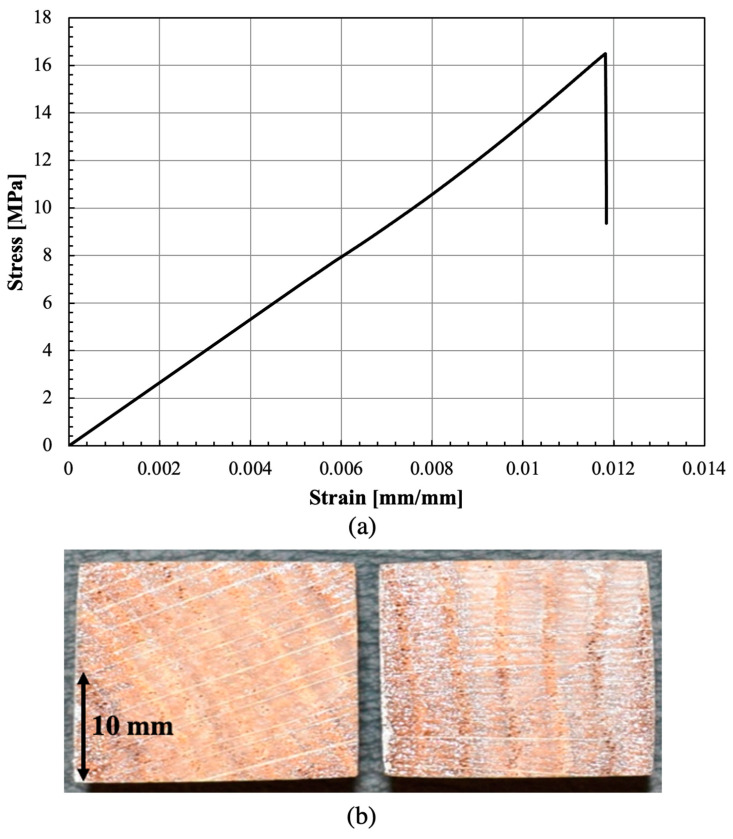
Representative tensile stress–strain curve of the bio-based adhesive (**a**), and fracture surface (**b**).

Figure 13a presents stress–strain curves for the bio-based adhesive using DIC to obtain the described. The Young’s modulus obtained was 1.32 ± 0.05 GPa, and the average tensile strength of the adhesive was 16.45 ± 0.55 MPa, values comparable to petroleum-based structural adhesives. To ensure the testing process’ accuracy, the fracture surfaces were meticulously analyzed to verify cohesive failure in the adhesive. This was confirmed by the presence of adhesive on top of both substrates. All samples exhibited visibly cohesive failure without any delamination, which indicates that the bio-based adhesive exhibited an appropriate bonding strength to the oak-wood substrates (see Figure 13b).

**Figure 14:**
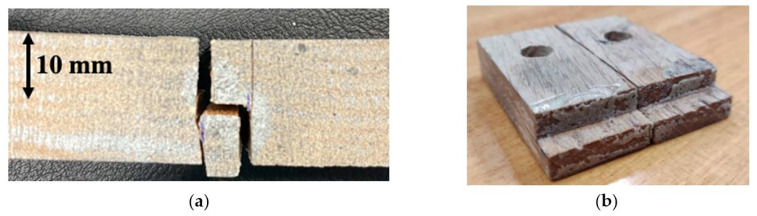
Fracture surface of TAST specimen: substrate loaded in transverse direction (**a**); substrate loaded in fiber direction (**b**).

During the testing process aimed at determining the shear strength and modulus of the adhesive, consistent failure occurred across the wood substrate, resulting in either the wood breaking or delamination (Figure 14). Consequently, it became evident that accurately measuring the shear properties of the adhesive itself was not feasible through this method. The main challenge arose from the nature of the adhesive’s curing process, which solely takes place on wood surfaces. This leads to interpenetration between the adhesive and the wood substrate, contributing to the overall joint strength. As a result, it was not possible to isolate and accurately quantify the shear properties of the adhesive alone.

#### 4.2.2. Fracture Tests

**Figure 15:**
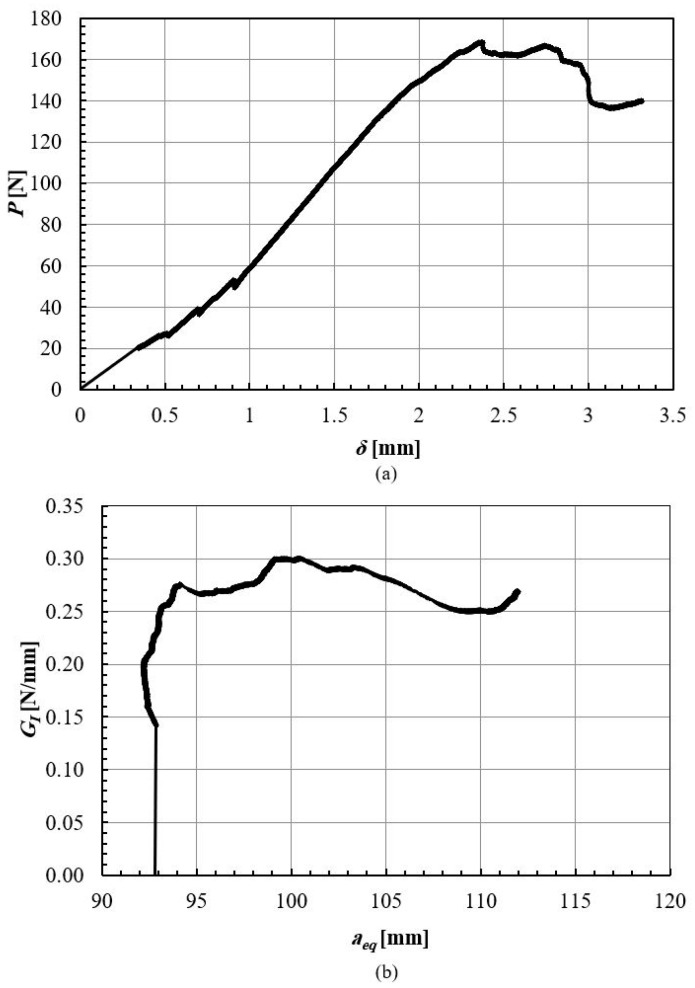
DCB test results for the adhesive: P-δ curve (**a**) and R-curve (**b**).

**Figure 16:**
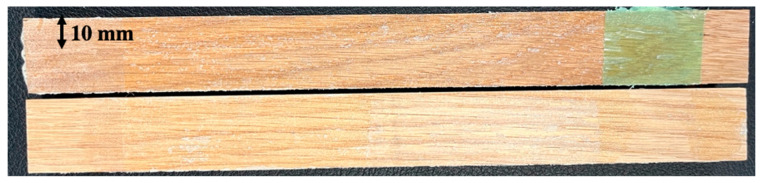
DCB specimens’ fracture surfaces.

The fracture toughness of the adhesive in mode I was obtained from the *P-δ* curve of the DCB test, shown in Figure 15a, and through applying CBBM and generating the R-curve, shown in Figure 15b. The average mode I fracture energy of the bio-based adhesive was 0.33 ± 0.03 N/mm. The samples exhibited cohesive failure, thus returning a fracture toughness representative of that of the adhesive layer, shown in Figure 16.

**Figure 17:**
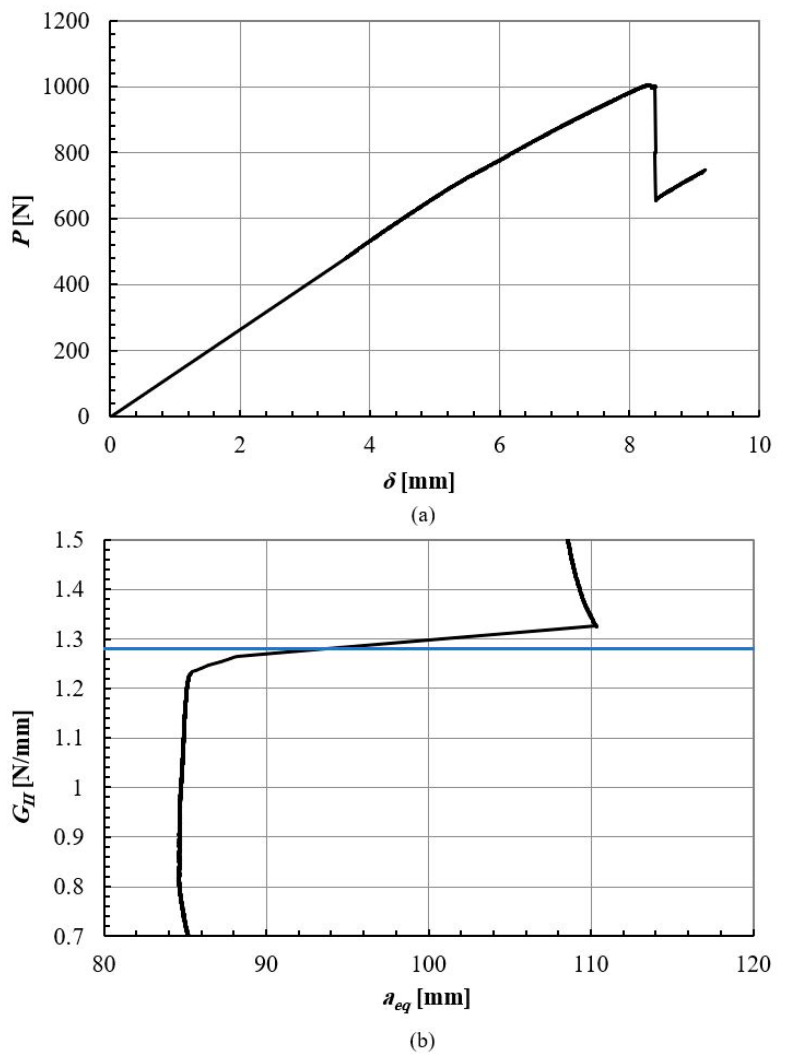
ELS test results for the adhesive: P-δ curve (**a**) and R-curve (**b**). Blue line presented the obtained fracture energy.

The same procedure was conducted for mode II from the *P-δ* curve of the ELS test, shown in Figure 17a, and through applying CBBM and obtaining the R-curve, shown in Figure 17b. The average mode II fracture energy was determined to be 1.2 ± 0.2 N/mm.

Typically, the average fracture energy for mode I fracture in commercial synthetic-urea formaldehyde adhesives falls within the range of 0.1 to 0.2 N/mm. For mode II fracture, this value extends between 0.2 to 0.4 N/mm [34,35]. In contrast, the bio-based adhesive showcased substantially elevated fracture energy values when compared to these conventional synthetic-urea–formaldehyde adhesives indicating its potential to enhance the durability and dependability of wooden products. In conclusion, the study highlights the promising potential of the bio-based adhesive as a sustainable alternative for the wood industry, offering superior fracture resistance and reliability for various fracture modes.

The cohesive properties of the bio adhesive are reviewed in Table 4.

The study’s results reveal the large potential of the studied bio-based polyurethane adhesive, which has demonstrated good strength and fracture energy for a bio-derived product. This performance positions it as a compelling choice for high-performance applications across multiple industries where sustainability is mandatory. This research also highlights the adhesive’s role as an eco-conscious alternative to traditional adhesives, driven by its outstanding bonding capabilities, environmentally friendly composition, and reduced emissions of harmful compounds. It is essential to note that the adhesive’s advantages extend beyond the study’s scope, encouraging future research to explore a wider array of applications and delve deeper into the molecular structure and formulation for further enhancements.

## 5. Conclusions

The study examined natural pine wood and its densified counterpart, which showed significant increases in density, stiffness, and strength (120%, 44%, and 85%, respectively). However, densification negatively impacted transverse properties due to lignin- and wood-matrix destruction. Nevertheless, densified wood remains promising for structural applications given the very large increases in strength and stiffness attained in the fiber direction.

The bio-based-adhesive characterization process revealed a significant tensile strength and cohesive failure, indicating strong adhesion to wood surfaces. Compared to synthetic adhesives, the bio-based adhesive exhibited high fracture energy values, offering a reliable and sustainable alternative. Overall, the combination of densified pine wood and the bio-based adhesive enhances mechanical properties and adhesion capabilities, with potential implications for various applications, improving performance, durability, and sustainability of wood-related products.

## Figures and Tables

**Table 1:** Elastic properties of pine wood determined by Olivera et al. [30].

*E_L_* [GPa]	*E_R_* [GPa]	*E_T_* [GPa]	*ν_LT_*	*ν_LR_*	*ν_TR_*	*G_LR_* [GPa]	*G_LT_* [GPa]	*G_TR_* [GPa]
12.0	1.9	1.0	0.5	0.4	0.3	1.1	1.0	0.3

**Table 2:** Strength properties of wood (W) and densified wood (D).

	*E*_Fiber direction_ [GPa]	*σ*_Fiber direction_ [MPa]	*E*_Transverse direction_ [MPa]	*σ*_Transverse direction_ [MPa]
W	12 ± 1	97.3 ± 8.3	155 ± 22	2.9 ± 0.2
D	31± 1	180.1 ±12.9	43 ± 3	2.1 ± 0.1

**Table 3:** Fracture properties of wood (W) and densified wood (D), along fiber direction (LR) and perpendicular to the fiber (RL).

	W_LR_	D_LR_	W_RL_	D_RL_
*K_IC_* [MPa/m]	17.7 ± 0.3	39.4 ± 1.5	24.0 ± 4.1	128.1 ± 15.1
*G_IC_* [N/mm]	0.20 ± 0.01	0.76 ± 0.09	0.33 ± 0.04	0.75 ± 0.10

**Table 4:** Bio-based-adhesive properties.

*E* [MPa]	*σ* [MPa]	*G_IC_* [N/mm]	*G_IIC_* [N/mm]
197.09 ± 9.76	3.27 ± 0.14	0.30 ± 0.03	1.27 ± 0.10

## Data Availability

Data are contained within the article.
